# Phytochemical profiling and pharmacological evaluation of *Fingerhuthia africana* lehm: radical-scavenging behavior, genotoxicity, and antidiarrheal activities

**DOI:** 10.3389/fphar.2026.1835978

**Published:** 2026-06-22

**Authors:** Hamid Ullah, Muhammad Adil, Chong Zhang

**Affiliations:** 1 Center of Legume Plant Genetics and System Biology, Oil Crops Research Institute, College of Agriculture, Fujian Agriculture and Forestry University (FAFU), Fuzhou, China; 2 Key Laboratory of Ministry of Education for Genetics, Breeding and Multiple Utilization of Crops, Key Laboratory of Biological Breeding for Fujian and Taiwan Crops, Ministry of Agriculture and Rural Affairs, Fujian Agriculture and Forestry University, Fuzhou, China; 3 Department of Chemical and Life Science, Qurtuba University of Science and Information Technology, Peshawar, Pakistan; 4 Department of Botany, University of Swat, Charbagh, Pakistan

**Keywords:** antidiarrheal, antioxidant, comet assay, *Fingerhuthia africana*, GC‒MS, phytochemicals

## Abstract

**Objective:**

*F. africana* Lehm (Poaceae) is a perennial grass traditionally used in ethnomedicine for the treatment of gastrointestinal disorders and wound healing. Despite its traditional relevance, its phytochemical composition and pharmacological activities remain largely unexplored. This study aimed to comprehensively characterize the phytochemical and elemental profiles of *Fingerhuthia africana* and evaluate its DPPH radical-scavenging behavior, genotoxicity, and antidiarrheal effects.

**Methods:**

Phytochemical profiling of the methanolic extract was performed using gas chromatography mass spectrometry (GC–MS), while elemental composition was determined by atomic absorption spectrophotometry. DPPH radical-scavenging behavior was assessed using the DPPH assay, genotoxic potential was evaluated through the alkaline comet assay in human lymphocytes, and antidiarrheal activity was investigated in Swiss albino mice using the charcoal meal gastrointestinal transit model.

**Results:**

GC–MS analysis identified 35 metabolites, including 2-cyclohexen-1-one derivatives, α-terpineol, bis(2-ethylhexyl) phthalate, and γ-sitosterol, which are associated with various biological activities. Elemental analysis revealed the presence of essential minerals, including calcium (12.4 mg/g), potassium (9.8 mg/g), and iron (0.45 mg/g). The methanolic extract exhibited concentration-dependent DPPH radical-scavenging behavior, reaching 79.5% scavenging at 300 μg/mL. However, concentration-dependent DNA damage was observed in the comet assay across the tested range of 500–1,000 μg/mL, with the highest response at 1,000 μg/mL. *In vivo* experiments demonstrated significant antidiarrheal activity, with the extract inhibiting intestinal transit by up to 83.78% at 300 mg/kg.

**Conclusion:**

These findings indicate that *F. africana* is a chemically diverse species with measurable radical-scavenging behavior and significant antidiarrheal effects *in vivo*. However, the concentration-dependent genotoxic effects observed at higher concentrations highlight the need for further mechanistic studies and comprehensive toxicological evaluation before therapeutic relevance can be established.

## Graphical Abstract

**Figure:**
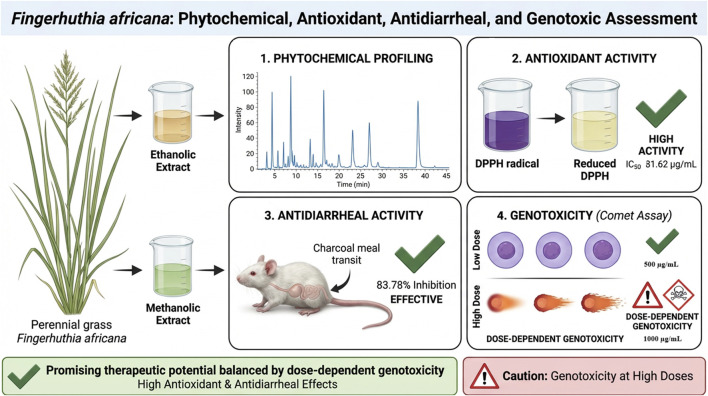

## 1 Introduction

Medicinal plants have been foundational to both traditional and modern healthcare systems, with increasing global interest in their therapeutic potential (Jamal, 2023; Manisha et al., 2025). It is estimated that approximately 80% of the population in developing countries relies on botanical drugs remedies, which are valued for their accessibility, affordability, and cultural relevance (Goldman and Nagra, 2026; Smith-Hall et al., 2012). These plants serve as a rich source of bioactive metabolites such as alkaloids, flavonoids, tannins, and terpenoids (Awuchi, 2020; Bagchi et al., 2025; Dar et al., 2023; Nwozo et al., 2023; Roy et al., 2022). As modern pharmacological research progresses, the bioactivity of these metabolites is becoming more widely recognized, opening new possibilities for their use in drug development (El-Saadony et al., 2025; Latif and Nawaz, 2025).

In Pakistan, the plant kingdom is particularly diverse, with approximately 6,000 plant species, of which 400–600 have been documented for their traditional medicinal uses. Despite this rich botanical heritage, only a small fraction of the native flora has been scientifically evaluated for its pharmacological potential (Muhammad et al., 2025; Sultana et al., 2025). Advances in analytical techniques such as gas chromatography-mass spectrometry (GC-MS) and atomic absorption spectrophotometry (AAS) have provided powerful tools for identifying the metabolites and essential minerals that contribute to the pharmacological effects of plants, yet many species remain unexplored in this context (Mutha et al., 2025; Nagori et al., 2025).

Several medicinal plants have been extensively investigated for their DPPH radical-scavenging capacity and gastrointestinal protective activities. Plant-derived metabolites, such as terpenoids, phenolics, and flavonoids, exhibit significant free radical-scavenging properties and demonstrate promising antidiarrheal effects in experimental models. These bioactive metabolites often contribute to the therapeutic efficacy of traditional botanical drug remedies used for digestive disorders (Akbari et al., 2022; Afsana Khan et al., 2025; Palombo, 2006).

*Fingerhuthia africana* Lehm (family: Poaceae), commonly known as thimble grass or Zulu fescue, is a perennial grass native to southern Africa, with populations also found in Khyber Pakhtunkhwa, Pakistan. Traditionally, *Fingerhuthia africana* has been used as fodder, but members of the Poaceae family are also reported to possess significant pharmacological activities such as anti-inflammatory, antibacterial, genotoxic and antidiarrheal effects (Artico et al., 2020; Majeed et al., 2020; Samreen and Shabeena, 2013; Swami et al., 2024). Despite the growing pharmacological interest in medicinal plants, *F. africana* remains largely unexplored, and its metabolites composition and biological activities have not yet been systematically investigated, thus presenting a significant gap in the scientific literature.

Given this gap, the present study was undertaken to characterize the metabolites profile of *F. africana* using GC–MS to provide insight into its metabolites composition (Ralte et al., 2022), determine its elemental and proximate composition (Shahid et al., 2023), and evaluate its DPPH radical-scavenging behavior, genotoxicity, and antidiarrheal effects using established analytical, *in vitro*, and *in vivo* methods (Agnihotri et al., 2025; Laaraj et al., 2025; Noor and Terefe, 2025). Collectively, these analyses provide the first integrated scientific assessment of this species and help connect its traditional use with experimental evidence.

## 2 Methods

### 2.1 Chemicals and reagents

All chemicals and reagents used in this study were of analytical grade and obtained from Sigma-Aldrich (Germany). These included methanol, ethanol, nitric acid (HNO_3_), perchloric acid (HClO_4_), sulfuric acid (H_2_SO_4_), sodium hydroxide (NaOH), boric acid (H_3_BO_3_), hydrochloric acid (HCl), petroleum ether, copper sulfate, potassium sulfate, hydrogen peroxide (H_2_O_2_), ethidium bromide, and 2,2-diphenyl-1-picrylhydrazyl (DPPH). The materials used in the *in vivo* assays, including castor oil, atropine sulfate, charcoal powder, and gum acacia, were also purchased from Sigma-Aldrich (Germany). Data analysis was performed using SPSS version 20.0 (IBM, United States) and GraphPad Prism version 8.0.2 (GraphPad Software, United States).

### 2.2 Plant collection and identification

Fresh samples of *F. africana* Lehm were collected from Mohabati Killa village, Karak District, Khyber Pakhtunkhwa, Pakistan (32°48′–33°23′N, 70°40′–71°30′E) during July–August 2023. The climate of the area is semi-arid, with an annual average rainfall of approximately 450 mm, and temperatures ranging from 45 °C during the summer months to 10 °C in winter (Asif Khan et al., 2025). The predominant soil types in the region are sandy loam and clay loam, which exhibit alkaline pH levels and moderate fertility, making them suitable for agriculture and grazing (Zamin et al., 2025). Taxonomic identification was conducted following the guidelines in the *Flora of Pakistan* (Stewart et al., 1972). A voucher specimen (H. Ullah Bot. 1,622) was deposited at the Herbarium of the Department of Chemical and Life Sciences, Qurtuba University, Peshawar, Pakistan.

### 2.3 Preparation of extracts

The collected plant material was thoroughly washed, shade-dried, and ground into a fine powder using a mechanical grinder. For extraction, 100 g of the powdered material was separately macerated in 1 L of methanol and 1 L of 80% ethanol (v/v) for 72 h at 180 rpm. These solvents were selected because their differing polarities allow broader extraction of metabolites and facilitate comparison of the resulting biological responses. In particular, aqueous ethanol is widely used in medicinal plant studies for the recovery of relatively polar bioactive metabolites (Song and Lee, 2015; Vo & Nguyen, 2023). After extraction, each mixture was filtered, and the respective filtrates were concentrated under reduced pressure using a rotary evaporator (RE201D, Biobase, China) at 40 °C. The concentrated extracts were further dried in a water bath and stored at 4 °C until subsequent analysis and biological evaluation (Arundina et al., 2025; Zhu et al., 2025).

### 2.4 Gas chromatography-mass spectrometry (GC–MS) analysis

Metabolite profiling of the methanolic extract of *F. africana* was performed using an Agilent 7890B gas chromatograph coupled with an Agilent mass spectrometer. Before analysis, the dried extract was re-dissolved in methanol and analyzed directly without derivatization. A 1 µL aliquot was introduced in splitless mode onto a DB-5MS capillary column (30 m × 0.25 mm, 0.15 µm film thickness). Helium served as the carrier gas at a constant flow rate of 1 mL/min. The oven temperature was initially maintained at 55 °C for 0.4 min, then increased to 200 °C at a rate of 25 °C/min, followed by a rise to 280 °C at 8 °C/min, and finally to 300 °C at 25 °C/min, where it was held for 2 min. Putative metabolite identification was achieved by comparing recorded mass spectra with the NIST mass spectral library, using retention index information where applicable to support the assignments. Since the extract was analyzed without derivatization, the resulting profile primarily reflects volatile and semi-volatile metabolites amenable to direct GC–MS analysis (Sujay and Balaji, 2025; Thamer and Thamer, 2023).

### 2.5 Elemental analysis

Elemental analysis was performed by digesting 0.5 g of dried plant powder with 10 mL of HNO_3_ for 24 h. Afterwards, 4 mL of HClO_4_ was added, and the mixture was heated until white fumes appeared. The solution was then cooled, diluted to 100 mL with deionized water, and filtered. Elemental concentrations of Ca, Mg, K, Na, Cu, Pb, Zn, Mn, Fe, Cd, and Co. were determined using atomic absorption spectrophotometry (AAS) as per the method outlined by (Bankaji et al., 2023).

### 2.6 Proximate analysis

Proximate composition of the plant parts (roots, stems, and leaves) was determined according to AOAC guidelines (AOAC, 1990; Bankaji et al., 2023). Moisture content was measured by drying 2 g of the sample at 105 °C until a constant weight was achieved. Ash content was determined by incinerating 2 g of dried sample at 600 °C in a muffle furnace. Crude fat was extracted using petroleum ether (40 °C–60 °C) in a Soxhlet apparatus for 3 h. Crude protein was estimated by the Kjeldahl method with a conversion factor of N × 6.25. Crude fiber was determined after sequential acid and alkali digestion, followed by ashing. Carbohydrate content was calculated by difference: 100 – (moisture + ash + fat + protein + fiber). Gross energy was estimated using Atwater conversion factors (4 kcal/g for protein and carbohydrate, 9 kcal/g for fat) (Ismail, 2024; Nwosu et al., 2022).

### 2.7 Genotoxicity assay (comet assay)

The genotoxic potential of the plant extracts was evaluated using the alkaline comet assay (Collins et al., 2023; Yıldız et al., 2025) with some modifications. Human lymphocytes were isolated and treated with plant extracts at concentrations of 500, 750, and 1,000 μg/mL. Hydrogen peroxide (H_2_O_2_, 100 µM for 30 min) was used as the positive control. The cells were embedded in low-melting agarose on pre-coated slides, lysed, and electrophoresed under alkaline conditions (pH > 13). The DNA was stained with ethidium bromide, and the slides were examined under a fluorescence microscope. DNA damage was quantified based on tail length and total comet score (TCS).

### 2.8 DPPH radical-scavenging capacity (chemical assay)

Antioxidant activity was assessed using the DPPH radical scavenging assay, as described by (Baliyan et al., 2022) with minor modifications. Extracts (100, 200, and 300 μg/mL) were mixed with 0.135 mM DPPH in methanol (1:1 v/v). The reaction mixture was incubated in the dark at room temperature for 30, 60, and 90 min. Absorbance was measured at 517 nm using a UV-Vis spectrophotometer. Ascorbic acid was used as the reference standard. Radical scavenging activity (%) was calculated as:

$$\%\text{ RSA}=\left[\left(\text{AC}—\text{AS}\right)/\text{ AC}\right]\times 100$$

Where ‘Ac’ is the absorbance of the control and ‘As’ is the absorbance of the sample.

### 2.9 Antidiarrheal activity (charcoal meal test in mice)

Antidiarrheal activity was evaluated using the charcoal meal gastrointestinal transit test, as described by (Ayalew et al., 2022), with minor modifications. Albino mice (*n* = 25) were randomly divided into five groups (*n* = 5 per group). Each mouse was orally administered 1 mL of castor oil. After 1 h, the animals received the following treatments: Group I, normal saline (10 mL/kg, i. p.); Group II, atropine sulfate (10 mg/kg, i. p.); and Groups III–V, methanolic extract of *F. africana* at 100, 200, and 300 mg/kg, respectively, by the i. p. route. The intraperitoneal route was used as an initial proof-of-concept pharmacological approach to assess systemic antimotility effects while reducing variability associated with oral absorption and formulation-related factors (Al Shoyaib et al., 2020; Gakunga et al., 2013). Atropine sulfate was used as an antimotility reference drug because the charcoal meal test primarily measures gastrointestinal propulsion. As a muscarinic receptor antagonist, atropine reduces cholinergic stimulation of intestinal smooth muscle and thereby inhibits intestinal peristalsis (Al-Harbi et al., 2016; Marks et al., 2013; Sohji et al., 1978). After 1 h, a charcoal meal (10% charcoal in 5% gum acacia) was administered. Mice were euthanized 1 h later, and the distance travelled by the charcoal was measured relative to the total intestinal length. Intestinal transit (%) was calculated as:

$$\text{Intestinal transit }\left(\%\right)=\left(D/L\right)\;x\;100$$

where “D” represents the distance covered by the charcoal (in meters) and “L” is the intestinal length (in meters).

### 2.10 Ethical approval

The animal study was reviewed and approved by the Ethical Committee of Pharmacy Lab, University of Swat, Pakistan, under permit no (148/VIEC/VRIP).

### 2.11 Statistical analysis

All data are presented as mean ± standard deviation (SD). *In vitro* experiments (DPPH and comet assays) used (n = 3) biological replicates (independent days with fresh extracts and cells), each with technical duplicates. Elemental and proximate analyses used (n = 3). The antidiarrheal study used (n = 5) mice per group. Statistical significance was assessed by one-way ANOVA with Tukey’s HSD *post hoc* test (p < 0.05). Analyses used SPSS v20.0 and GraphPad Prism v8.0.2.

## 3 Results

### 3.1 Metabolites composition (GC–MS analysis)

**FIGURE 1:**
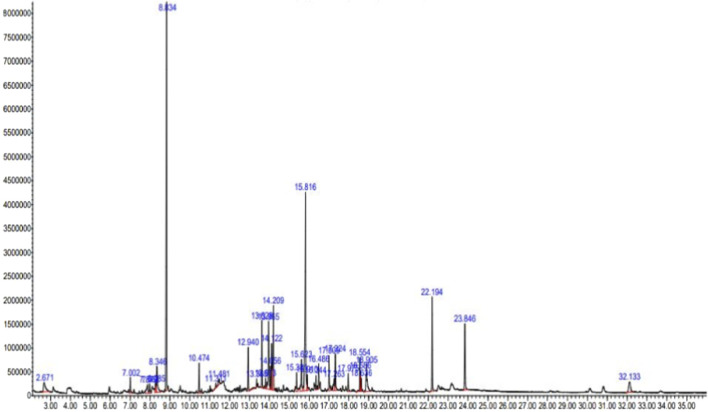
GC-MS chromatogram of the methanolic extract of *F*. *africana* Lehm, showing the identified metabolites and their corresponding retention times.

The methanolic extract of *F*. *africana* exhibited a complex GC–MS profile, with 35 metabolites detected across the chromatogram (Figure 1). The profile was dominated by terpenoid-related metabolites, fatty acid derivatives, aromatic metabolites, and sterol-like metabolites, indicating substantial chemical diversity within the extract. Among the detected metabolites, 2-cyclohexen-1-one, 3-methyl-6-(1-methylethyl)- (23.69%), acetic acid, (3-fluorophenyl) methyl ester (14.92%), and bis(2-ethylhexyl) phthalate (4.42%) were the most abundant based on relative peak area. In total, the detected metabolites represented 99.78% of the overall chromatographic area. A comprehensive list of the identified metabolites, together with their RT, relative abundance, MF, MW, and library matching probability, is presented in (Table 1). The most prominent peaks were observed at retention times of 8.834, 15.816, and 23.846 min, corresponding to the major detected metabolites described above, which may contribute to the biological properties investigated in this study.

**TABLE 1:** List of major metabolites identified in the methanolic extract of *F. africana* using GC-MS analysis.

S. no	RT	%Area	Name of metabolite	MF	MW (g/mol)	Prob. %
1	2.671	1.94	1,3-Dihydroxyacetone dimer	C_3_H_6_O_3_	90.08	64
2	7.002	0.99	4H-Pyran-4-one, 2,3-dihydro-3,5-dihydroxy-6-methyl-	C_6_H_8_O_4_	144.13	96
3	7.879	1.17	4-Methyl itaconate	C_6_H_8_O_4_	144.13	43
4	7.982	0.53	α-Terpineol	C_10_H_18_O	154.24	91
5	8.285	0.93	1,2,3-Propanetriol, 1-acetate	C_5_H_10_O_4_	134.13	37
6	8.346	2.52	Benzofuran, 2,3-dihydro-	C_8_H_8_O	120.15	95
7	8.834	23.69	2-Cyclohexen-1-one, 3-methyl-6-(1-methylethyl)-	C_10_H_16_O	152.23	97
8	10.474	1.59	p-tert.-Butylcatechol	C_10_H_14_O_2_	166.22	70
9	11.481	0.64	2-Hydroxy-3-methylbenzaldehyde	C_8_H_8_O_2_	136.15	46
10	12.94	2.06	Cyclohexanemethanol, 4-ethenyl-α,α,4-trimethyl-3-(1-methylethenyl)-, [1 R-(1α,3α,4β)]-	C_15_H_26_O	222.37	91
11	13.365	0.39	Caryophyllene oxide	C_15_H_24_O	220.35	81
12	13.628	3.09	α-epi-7-epi-5-Eudesmol	C_15_H_26_O	222.36	99
13	13.813	0.54	2-((2S,4aR)-4a,8-Dimethyl-1,2,3,4,4a,5,6,7-octahydronaphthalen-2-yl) propan-2-ol	C_15_H_26_O	222.37	99
14	13.965	5.62	Cyclohexene, 6-ethenyl-6-methyl-1-(1-methylethyl)-3-(1-methylethylidene)-, (S)-	C_15_H_24_	204.35	64
15	14.056	1.1	Hinesol	C_15_H_26_O	222.37	91
16	14.122	3.09	2-Naphthalenemethanol, decahydro-α,α,4a-trimethyl-8-methylene-, [2 R-(2α,4aα,8aβ)]-	C_15_H_26_O	222.37	99
17	14.209	5.29	(1aR,3aS,7S,7aS,7bR)-1,1,3a,7-Tetramethyldecahydro-1H-cyclopropa [a]naphthalen-7-ol	C_15_H_26_O	222.37	83
18	15.381	1.2	7-(2-Hydroxypropan-2-yl)-1,4a-dimethyldecahydronaphthalen-1-ol	C_15_H_28_O_2_	240.38	93
19	15.623	2.26	1H-Indene, 1-ethylideneoctahydro-7a-methyl-, cis-	C_12_H_20_	164.29	89
20	15.816	14.92	Acetic acid, (3-fluorophenyl) methyl ester	C_9_H_9_FO_2_	168.16	25
21	15.91	0.92	4-Methyl-1,2-bis(trimethylsilyloxy)pentane	C_12_H_30_O_2_Si_2_	262.54	22
22	16.344	0.65	7 R,8R-8-Hydroxy-4-isopropylidene-7-methylbicyclo [5.3.1]undec-1-ene	C_15_H_25_O	220.35	86
23	16.486	1.31	6-(2-Hydroxypropan-2-yl)-4,8a-dimethyl-2,3,4,6,7,8-hexahydro-1H-naphthalen-1-ol, 1-acetate	C_17_H_28_O_3_	280.40	53
24	17.009	1.87	Hexadecanoic acid, methyl ester	C_17_H_34_O_2_	270.45	99
25	17.263	1.12	2,7:3,6-Dimethanonaphthalene, decahydro-	C_12_H_18_	162.27	25
26	17.324	2.24	n-Hexadecanoic acid	C_16_H_32_O_2_	256.42	99
27	17.975	1.16	trans-8-Methyl-1β-acetylhydrindane	C_12_H_20_O	180.29	38
28	18.554	1.64	9,12-Octadecadienoic acid (Z, Z)-, methyl ester	C_19_H_34_O_2_	294.47	99
29	18.586	0.87	9,12,15-Octadecatrienoic acid, methyl ester, (Z,Z,Z)-	C_19_H_32_O_2_	292.46	99
30	18.636	0.55	9-Octadecenoic acid (Z)-, methyl ester	C_19_H_36_O_2_	296.49	99
31	18.905	3.39	9,12,15-Octadecatrienoic acid, (Z,Z,Z)-	C_18_H_30_O_2_	278.43	99
32	22.194	4.42	Bis(2-ethylhexyl) phthalate	C_24_H_38_O_4_	390.55	91
33	23.846	4.11	1,4-Benzenedicarboxylic acid, bis(2-ethylhexyl) ester	C_24_H_38_O_4_	390.56	95
34	32.133	1.97	γ-Sitosterol	C_29_H_50_O	414.70	98
Total (%)		99.78	​	​	​	​

RT, retention time; MF, molecular formula; MW, molecular weight; Prob, Probability.

### 3.2 Elemental composition

The macro- and trace elements profile of *F. africana* was analyzed using atomic absorption spectrophotometry, revealing significant mineral content across its various plant parts. The leaves displayed the highest concentrations of calcium (37.80 mg/kg), iron (3.35 mg/kg), and manganese (0.97 mg/kg), suggesting their potential nutritional and therapeutic importance. In contrast, the stem exhibited elevated levels of sodium (32.51 mg/kg), potassium (19.02 mg/kg), and magnesium (6.72 mg/kg), indicating a role in maintaining ionic balance and possibly contributing to the plant’s overall physiological functions. Cadmium and cobalt were either not detected or present in trace amounts, confirming the minimal presence of potentially toxic elements. These findings underscore the suitability of *F. africana* for medicinal applications, with a favorable elemental composition (Table 2).

**TABLE 2:** Macroelements and trace elements composition (mg/kg) in the leaves, stems, and roots of *Fingerhuthia africana*.

Plant parts	Macronutrients (mg/kg)				Micronutrients (mg/kg)						
	Ca	Mg	K	Na	Cu	Pb	Zn	Mn	Fe	Co	Cd
Leaves	37.80 ± 0.12^a^	5.64 ± 0.29^b^	18.65 ± 0.19^b^	13.16 ± 1.87^c^	0.05 ± 0.09^a^	0.08 ± 0.06^b^	0.73 ± 0.02^c^	0.97 ± 0.03^a^	3.35 ± 0.06^a^	0.00 ± 0.00^a^	0.03 ± 0.02^a^
Stem	33.75 ± 0.58^b^	6.72 ± 0.49^a^	19.02 ± 0.26^a^	32.51 ± 1.23^a^	0.05 ± 0.05^a^	0.13 ± 0.1^a^	1.18 ± 0.01^a^	0.49 ± 0.05^b^	0.37 ± 0.09^c^	0.00 ± 0.00^a^	0.01 ± 0.01^b^
Roots	10.56 ± 0.20^c^	2.95 ± 0.73^c^	6.50 ± 0.27^c^	18.32 ± 1.73^b^	0.01 ± 0.03^b^	0.15 ± 0.03^a^	0.15 ± 0.01^b^	0.28 ± 0.02^c^	0.29 ± 0.04^b^	0.00 ± 0.00^a^	0.00 ± 0.00^c^

Values are expressed as mean ± SD (n = 3). Means in the same row followed by different letters are significantly different (p < 0.05, Tukey’s HSD, test).

### 3.3 Proximate composition

The proximate composition of the stems, leaves, and roots of *F. africana* was analyzed to assess its nutritional profile (Table 3). The highest moisture content was observed in the roots (8.57%), while the leaves exhibited the lowest moisture content (7.70%). Ash content was highest in the roots (9.77%) and lowest in the leaves (5.00%). Crude fat was most abundant in the roots (5.96%) and least in the stems (3.39%). The stem contained the highest proportion of crude fiber (45.03%), while the leaves had the lowest (39.00%). Crude protein was highest in the roots (3.62%) and lowest in the stems (2.92%). Carbohydrate content was highest in the leaves (39.71%) and lowest in the roots (30.31%). Finally, gross energy was greatest in the leaves (220.10 Kcal/100 g) and lowest in the stems (180.34 Kcal/100 g).

**TABLE 3:** Proximate composition of the leaves, stems, and roots of *F. africana*, including moisture, ash, fats, fiber, proteins, carbohydrates, and gross energy content (Kcal/100 g).

Plant. parts	Moisture (%)	Ash (%)	Fats (%)	Fibers (%)	Proteins (%)	Carbohydrates (%)	Gross energy (Kcal/100 g)
Leaves	7.70 ± 1.25^b^	5.00 ± 1.02^c^	5.38 ± 2.21^b^	39.00 ± 1.43^b^	3.21 ± 1.01^b^	39.71 ± 2.41^a^	220.10 ± 2.09^a^
Stem	8.09 ± 2.12^ab^	6.03 ± 1.03^b^	3.39 ± 1.19^c^	45.03 ± 1.15^a^	2.92 ± 1.12^c^	34.54 ± 1.06^b^	180.34 ± 1.35^c^
Roots	8.57 ± 2.01^a^	9.77 ± 2.01^a^	5.96 ± 2.03^a^	41.77 ± 3.51^b^	3.62 ± 1.04^a^	30.31 ± 1.89^c^	189.40 ± 3.11^b^

Values are expressed as mean ± SD (n = 3). Different superscript letters within a column indicate statistically significant differences among plant parts (one-way ANOVA, followed by Tukey’s HSD, test, p < 0.05). Values sharing the same letter are not significantly different.

### 3.4 Genotoxicity assay

#### 3.4.1 Methanolic extract

The genotoxicity of the methanolic extract of *F. africana* was assessed using the comet assay, revealing dose-dependent DNA damage in human lymphocytes. At a concentration of 1,000 μg/mL, the total comet score (TCS) was significantly elevated (85.13 ± 1.23), indicating a genotoxic effect. This was accompanied by a notable increase in comet classes 1–3 compared to the negative control, suggesting that higher concentrations of the extract induce DNA strand breaks and significant genotoxicity (Table 4). These findings underscore the potential for *F. africana* to affect genomic integrity at higher doses.

**TABLE 4:** Genotoxicity of the methanolic extract of *F. africana* in human lymphocytes (Comet assay).

Treatment	Class 0	Class 1	Class 2	Class 3	TCS
Negative Control	91.43 ± 1.52^a^	11.30 ± 0.00^e^	2.56 ± 0.47^c^	1.30 ± 0.00^c^	20.32 ± 0.65
Positive Control (H_2_O_2_, 100 μM, 30 min)	38.00 ± 2.30^e^	55.43 ± 1.07^a^	13.37 ± 0.48^b^	5.53 ± 1.07^a^	98.76 ± 2.18
500 μg/mL	88.42 ± 1.37^b^	13.53 ± 2.27^d^	3.43 ± 0.63^c^	4.38 ± 1.15^b^	33.53 ± 1.52^b^
750 μg/mL	85.47 ± 2.37^c^	18.27 ± 1.57^b^	1.56 ± 0.27^d^	3.26 ± 0.37^b^	31.17 ± 0.76^c^
1,000 μg/mL	75.67 ± 1.53^d^	15.66 ± 0.50^c^	26.44 ± 1.02^a^	5.53 ± 1.06^a^	85.13 ± 1.23^a^

Values represent the mean ± SD (n = 3), with statistical significance indicated by different superscript letters (p < 0.05, Tukey’s HSD, test).

#### 3.4.2 Ethanolic extract

The ethanolic extract of *F. africana* exhibited comparatively lower genotoxicity than the methanolic extract. At a concentration of 1,000 μg/mL, the total comet score (TCS) was significantly reduced (44.08 ± 1.40) compared to the methanolic extract (85.13 ± 1.23, Table 4). This suggests that the ethanolic extract exhibits a relatively safer genotoxic profile, with less DNA fragmentation and fewer observable changes in comet classes, indicating a lower degree of DNA damage (Table 5).

**TABLE 5:** Genotoxicity of the ethanolic extract of *F. africana* in human lymphocytes (Comet assay).

Treatment	Class 0	Class 1	Class 2	Class 3	TCS
Negative Control	88.00 ± 2.30^a^	8.54 ± 1.43^d^	6.35 ± 1.50^e^	4.43 ± 2.00^d^	34.53 ± 2.32
Positive Control (H_2_O_2_, 100 μM, 30 min)	35.63 ± 2.50^e^	40.50 ± 3.25^a^	27.26 ± 2.17^b^	16.30 ± 1.20^a^	143.80 ± 2.06
500 μg/mL	84.34 ± 2.50^b^	35.53 ± 1.41^b^	30.43 ± 2.50^a^	10.33 ± 2.50^b^	127.38 ± 1.16^a^
750 μg/mL	66.52 ± 3.20^c^	23.40 ± 2.00^c^	18.63 ± 2.50^c^	7.54 ± 1.10^c^	83.28 ± 0.65^b^
1,000 μg/mL	56.31 ± 2.13^d^	20.42 ± 3.60^c^	15.47 ± 2.10^d^	4.38 ± 1.00^d^	44.08 ± 1.40^c^

Values are presented as mean ± SD (n = 3), with statistical differences denoted by different superscript letters (p < 0.05, Tukey’s HSD test).

### 3.5 DPPH radical-scavenging capacity

**FIGURE 2:**
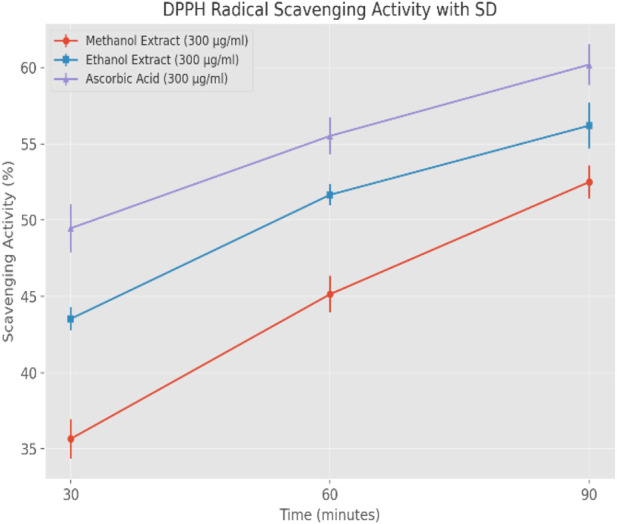
DPPH radical-scavenging capacity of *F. africana* extracts and ascorbic acid at 300 μg/mL. Values are mean ± SD (n = 3 biological replicates).

The DPPH radical scavenging capacity of both the methanolic and ethanolic extracts of *F. africana* was evaluated as part of the phytochemical-analytical characterization and is presented in (Figure 2). Both extracts exhibited a time- and concentration-dependent increase in their radical scavenging ability. At a concentration of 300 μg/mL after 90 min, the methanolic extract demonstrated the highest antioxidant activity (79.5%) compared to the ethanolic extract (76.5%). However, both extracts showed lower radical-scavenging capacity than the ascorbic acid control (91.5%).

### 3.6 Antidiarrheal activity

**FIGURE 3:**
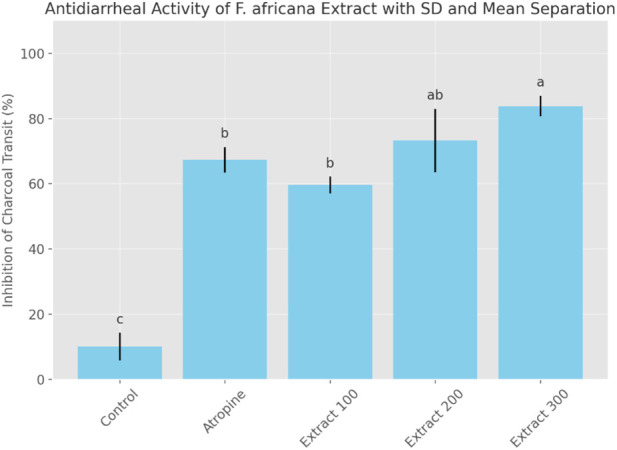
Antidiarrheal activity of the methanolic extract of *F. africana* in mice. Bars represent mean ± SD (*n* = 5). Different superscript letters above the bars indicate statistically significant differences among treatment groups, as determined by one-way ANOVA followed by Tukey’s HSD *post hoc* test (*p* < 0.05).

Based on the phytochemical profiling and *in vitro* biological screening, the methanolic extract of *F. africana* was selected for evaluation of *in vivo* antidiarrheal activity using the charcoal meal test. The extract exhibited a dose-dependent inhibition of intestinal transit (Table 6; Figure 3). The highest inhibition (83.78%) was observed at 300 mg/kg, which significantly exceeded that of the positive control (atropine sulfate, 67.37%). The 200 mg/kg and 100 mg/kg doses showed 73.26% and 59.63% inhibition, respectively. The control group (normal saline) demonstrated minimal inhibition (10.09%). Statistical analysis (one-way ANOVA, Tukey’s HSD test) confirmed significant differences between the 300 mg/kg dose and the control and atropine sulfate (p < 0.05).

**TABLE 6:** Antidiarrheal activity of crude methanolic extract of *F. africana*.

Treatment	Dose (mg/kg)	Mean length of intestine (cm)	Mean distance travelled by charcoal (cm)	Transit (%)	Percent inhibition (%)
Normal saline	10 mL/kg	54.8	49.27 ± 4.25	89.90%	10.09%^c^
Atropine sulfate	10 mg/kg	55.42	18.08 ± 3.93	32.62%	67.37%^b^
Methanolic Extract	100 mg/kg	51.83	20.92 ± 2.54	40.36%	59.63%^b^
	200 mg/kg	51.35	13.73 ± 9.72	26.73%	73.26%^ab^
	300 mg/kg	51.68	8.37 ± 3.16	16.21%	83.78%^a^

Values are presented as mean ± SD (*n* = 5). Percent inhibition was calculated from the charcoal transit distance relative to the control group. Different superscript letters indicate statistically significant differences among groups according to one-way ANOVA, followed by Tukey’s HSD, *post hoc* test (*p* < 0.05); groups sharing at least one letter are not significantly different.

These results suggest that the methanolic extract of *F. africana* exhibits potent antidiarrheal properties, with the 300 mg/kg dose outperforming atropine sulfate. Further studies are needed to validate these findings.

## 4 Discussion

The present study provides the first comprehensive metabolites and pharmacological characterization of *F. africana*, integrating GC–MS profiling (Table 1; Figure 1), radical-scavenging assessment (Figure 2), genotoxicity screening (Tables 4, 5), and *in vivo* antidiarrheal evaluation (Table 6; Figure 3). Collectively, the results indicate that *F. africana* contains bioactive metabolites with measurable radical-scavenging behavior and gastrointestinal effects, while also highlighting dose-dependent safety considerations.

GC–MS analysis revealed a chemically diverse profile dominated by terpenoids/sesquiterpenoids, fatty acid derivatives, aromatic metabolites, and phytosterols (Table 1; Figure 1). These chemical classes are frequently associated with radical-scavenging, anti-inflammatory, antimicrobial, and gut-modulating activities in medicinal plants (Table 7) (Kalasariya et al., 2023; Krishnan et al., 2024; Ma et al., 2025). Notably, α-terpineol identified in the extract has been experimentally shown to exert antidiarrheal activity in rodent models, supporting the plausibility that monoterpenoids in *F. africana* may contribute to reduced intestinal transit and improved gut function (Chen et al., 2023; Wu et al., 2023). In addition, caryophyllene oxide and related sesquiterpenoids have been discussed in the context of antidiarrheal activity through anti-inflammatory and antimicrobial pathways that can reduce intestinal irritation and pathogen-driven diarrhoea, which aligns with the broader phytochemical logic of the present findings (Damtie, 2023). Phytosterols such as γ-sitosterol may further support gastrointestinal relevance through anti-inflammatory effects and mucosal protective roles reported for phytosterols in digestive conditions (Miszczuk et al., 2024).

**TABLE 7:** List of selected GC–MS metabolites in *F. africana* based on reported bioactivities.

Metabolite	Chemical class	Reported biological activities	References
α-Terpineol	Monoterpenoid alcohol	Antimotility/antidiarrheal/anti-inflammatory	dos Santos Negreiros et al. (2019)
Caryophyllene oxide	Sesquiterpenoid	Anti-inflammatory/antimicrobial	Gertsch et al. (2008), Legault and Pichette (2007)
γ-Sitosterol	Phytosterol	Anti-inflammatory/gastrointestinal protective	Bouic (2001)
Hexadecanoic acid methyl ester	Fatty acid ester	Radical-scavenging/membrane-active effects	Aparna et al. (2012)
n-Hexadecanoic acid (palmitic acid)	Fatty acid	Anti-inflammatory/lipid-mediated signaling	Aparna et al. (2012)
Linoleic acid derivatives	Polyunsaturated fatty acids	radical-scavenging/anti-inflammatory	Das (2006)
2-Cyclohexen-1-one derivatives	Aromatic/ketone compounds	radical-scavenging/antimicrobial/cytotoxic activity	Džambić et al. (2020)
9,12,15-Octadecatrienoic acid	Fatty acid	radical-scavenging/anti-Cancer/anti-inflammatory	Parvathi et al. (2022)
Bis(2-ethylhexyl) phthalate	Phthalate ester	Genotoxic/Antibacterial	Javed et al. (2022)
1,4-Benzenedicarboxylic acid, bis(2-ethylhexyl) ester	Phthalate ester	Genotoxic/Phytotoxic	Lee et al. (2012)

In addition to metabolite diversity, both extracts demonstrated concentration- and time-dependent DPPH radical-scavenging capacity (Figure 2). While the methanolic extract showed slightly higher maximal inhibition at the highest tested concentration, the ethanolic extract exhibited a lower IC_50_, indicating greater apparent potency. Solvent-dependent differences in radical-scavenging capacity are widely reported in phytochemical studies and are commonly attributed to differences in extraction efficiency for phenolic and moderately polar metabolites, including phenolics and oxygenated terpenoids, which are often extracted efficiently in ethanol-based systems (Hossain et al., 2024; Miszczuk et al., 2024). These findings are consistent with reports on other Poaceae species, such as *Cymbopogon citratus* and *Setaria italica*, which also possess polyphenolic antioxidants with free-radical-scavenging properties (Dar et al., 2022; Łukaszyk et al., 2024; Wahyuni et al., 2024). Mechanistically, the DPPH model reflects electron or hydrogen donation capacity, suggesting that the antioxidant action of *F. africana* extracts may involve direct radical neutralization and interruption of oxidative chain reactions, even though both extracts remained less potent than the reference standard (Zahra et al., 2024).

Genotoxicity screening via comet assay (Tables 4, 5) indicated an overall increase in DNA damage, which was most pronounced in the methanolic extract at the highest tested concentration. However, the methanolic extract did not show a strictly monotonic pattern at the two lower concentrations, as the TCS at 750 μg/mL was slightly lower than that at 500 μg/mL. Such non-monotonic responses are recognized in toxicological datasets and should not be dismissed solely because they do not follow a classic linear dose–response trend. In complex botanical extracts, this type of pattern may reflect concentration-dependent shifts in redox behaviour, whereby metabolites with antioxidant properties at lower exposure can exert pro-oxidant or DNA-damaging effects at higher concentrations, particularly in sensitive cellular systems (Hill et al., 2018; Lewandowska et al., 2026). In addition, comet assay readouts can be influenced by cytotoxic or apoptosis-related processes, which may contribute to apparently non-linear responses and therefore require cautious interpretation (Collins et al., 2023; Cordelli et al., 2021). Because no parallel cytotoxicity assay was performed in the present study, the basis of this deviation cannot be determined conclusively and should be interpreted with caution. Importantly, because the comet assay in the present study was conducted using crude extracts rather than isolated fractions or purified metabolites, it was not possible to directly identify the specific metabolites responsible for the observed DNA damage. The genotoxic response, therefore likely reflects the combined action of multiple metabolites within the extract, and definitive identification of the responsible metabolites will require bioactivity-guided fractionation followed by metabolite-specific genotoxicity testing (Bucar et al., 2013). Comparable genotoxicity assessments in medicinal plant research have likewise emphasized the importance of defining an appropriate therapeutic window and undertaking follow-up toxicological evaluation rather than interpreting extract-level responses in isolation (Jalilzadeh-Amin & Maham, 2015). The comparatively lower genotoxic response observed with the ethanolic extract further suggests that the extraction solvent may influence the abundance of DNA-reactive constituents or modify the balance between protective and potentially damaging metabolites. However, because phytochemical profiling was performed only for the methanolic extract, the compositional basis for the differential genotoxic responses of the methanolic and ethanolic extracts could not be directly established in the present study; this will require dedicated comparative chemical profiling and, ideally, bioactivity-guided fractionation in future work.

The *in vivo* charcoal meal test demonstrated that the methanolic extract reduced intestinal transit in a dose-related manner (Table 6; Figure 3). Atropine sulfate was used in this model as an antimotility reference rather than as a broad antidiarrheal standard, because its antimuscarinic action suppresses cholinergic stimulation of intestinal smooth muscle and reduces gastrointestinal propulsion. Findings where plant extracts show comparable or strong antimotility effects relative to atropine have been reported in the literature; for example, the methanolic extract of the medicinal grass *Cynodon dactylon* demonstrated significant antidiarrheal activity and was reported as comparable to atropine sulfate in experimental models (Babu et al., 2009). Such outcomes are biologically plausible because botanical extracts can act through multiple pathways simultaneously rather than a single receptor target. In the context of *F. africana*, the antimotility effect may reflect combined mechanisms, including attenuation of smooth muscle contraction (e.g., via calcium influx modulation), reduced enteric excitability, and suppression of inflammatory mediators that amplify motility and secretion (Docsa et al., 2022). This mechanistic explanation is consistent with evidence that monoterpenoids such as α-terpineol can reduce diarrheal output and motility in rodents, supporting a direct pharmacodynamic link between identified metabolites (Tables 1, 7) and the observed physiological effect (dos Santos Negreiros et al., 2019). Moreover, literature on antidiarrheal medicinal plants repeatedly highlights that effective extracts often reduce intestinal propulsion and secretion concurrently, reflecting multi-target activity rather than pure antimuscarinic action (Sadraei et al., 2011; Woldeab et al., 2018).

Elemental and proximate analyses (Tables 2, 3) add useful context for safety and nutritional relevance. The presence of essential macroelements (Ca, K, Mg) and trace elements (Fe, Zn), alongside negligible toxic metal levels, supports a favourable elemental profile. These trace elements are essential for metabolic regulation and immune function, and their presence supports the traditional dietary use of grasses in rural populations (López-Alonso et al., 2025; Shao et al., 2021). While nutritional composition is not the primary driver of acute pharmacological effects, Fiber and mineral content may contribute indirectly to gastrointestinal health and resilience, and the low heavy metal burden strengthens the baseline safety argument for further development (Mititelu et al., 2025; Román-Ochoa et al., 2024).

This study has several limitations that should be acknowledged. First, only the methanolic extract was advanced to the *in vivo* antidiarrheal model, and the extract was administered intraperitoneally rather than orally. Although intraperitoneal administration can serve as an initial pharmacological screening route to assess systemic effects on intestinal transit, it does not replicate traditional oral use or direct luminal exposure in the gastrointestinal tract. Therefore, the present findings should be interpreted as preliminary evidence of antimotility activity, and future studies should repeat the antidiarrheal evaluation using oral administration. Second, no receptor-level or pathway-specific experiments were performed to verify the underlying mechanism, such as muscarinic antagonism, calcium channel modulation, antisecretory activity, or prostaglandin-related effects. Third, the concentration-dependent DNA damage observed in the comet assay indicates that broader safety evaluation is required, including sub-acute toxicity studies, histopathological assessment, and oxidative stress biomarkers to better define a safer exposure range. In addition, the *in vitro* genotoxic concentrations used in the comet assay (500–1,000 μg/mL, whole extract) cannot be directly compared with the *in vivo* antidiarrheal doses (100–300 mg/kg), because systemic exposure after intraperitoneal administration is governed by absorption, distribution, metabolism, and elimination. Although a simple theoretical estimate based on mouse blood volume suggests that the highest administered dose could correspond to a nominal whole-extract concentration above the *in vitro* range if it were entirely confined to blood, this scenario is not physiologically realistic (Al Shoyaib et al., 2020). Therefore, it remains uncertain whether the genotoxic concentrations observed *in vitro* are actually achieved *in vivo*, and this question will require dedicated pharmacokinetic investigation (Benet & Sodhi, 2022; Hines et al., 2022). Future studies should also prioritize comparative metabolites profiling, fractionation, and identification of the most active constituents, followed by mechanistic validation in complementary diarrheal models, including castor oil-induced diarrhea, enteropooling, and spasmolytic assays.

Overall, the findings support *F. africana* as a biologically active species with demonstrable radical-scavenging capacity and strong antidiarrheal effects that are consistent with the presence of terpenoid- and lipid-derived metabolites (Tables 1, 7). At the same time, dose-dependent genotoxicity underscores the need for careful safety evaluation and mechanistic clarification before therapeutic translation.

## 5 Conclusion

The present study provides the first integrated scientific assessment of *F. africana*, demonstrating that this species possesses a chemically diverse GC–MS-detectable metabolite profile together with notable DPPH radical-scavenging behavior, dose-dependent antidiarrheal effects, and concentration-dependent genotoxicity *in vitro*. The methanolic extract showed pronounced inhibition of intestinal transit, while both methanolic and ethanolic extracts exhibited measurable radical-scavenging capacity. At the same time, the genotoxic effects observed at higher concentrations indicate that the biological potential of *F. africana* must be interpreted alongside important safety considerations. Overall, these findings support the value of *F. africana* as a promising source of biologically relevant metabolites, while also highlighting the need for further compound-level characterization, mechanistic studies, and comprehensive toxicological evaluation before any therapeutic application can be considered.

## Data Availability

The datasets presented in this study can be found in online repositories. The names of the repository/repositories and accession number(s) can be found in the article/supplementary material.
